# Effect of Contents on the Electrical and Piezoelectric Properties of (1 − x)(Bi, Na)TiO_3_–x(Ba, Sr)TiO_3_ Lead-Free Piezoelectric Ceramics

**DOI:** 10.3390/ma16041469

**Published:** 2023-02-09

**Authors:** Seok-Mo Kang, Tae Wan Kim, Nam-Hoon Kim, Sung-Jin Kim, Jung-Hyuk Koh

**Affiliations:** 1 School of Electrical and Electronic Engineering, Chung-Ang University, Seoul 06974, Republic of Korea; 2 Department of Intelligent Energy and Industry, Chung-Ang University, Heukseok-ro, Seoul 06974, Republic of Korea; 3 Department of Electrical Engineering, Chosun University, Chosundae-gil, Dong-gu, Gwangju 61452, Republic of Korea; 4 College of Electrical and Computer Engineering, Chungbuk National University, Cheongju 28644, Republic of Korea

**Keywords:** piezoelectric materials, lead-free, ceramics, BNT–BST, electrical properties

## Abstract

In this study, the composition of lead-free piezoelectric ceramics (1 − x)(Bi_0.5_Na_0.5_)TiO_3_–x(Ba_0.5_Sr_0.5_)TiO_3_ with excellent piezoelectric properties was investigated. Crystal analysis and electrical and piezoelectric properties were analyzed according to the content of the BST composition. A phase change from rhombohedral to tetragonal structure was observed in 0.12 BST, and the densest and most uniform microstructure was confirmed in this composition. The dielectric constant increased from 905 to 1692 as the composition of BST increased to 0.12 BST. Afterward, as the composition of BST increased, the permittivity tended to decrease. Additionally, at 0.12 BST, P_r_ was the highest at 23.34 μC/cm^2^. The piezoelectric charge constant (d_33_) and the electromechanical coupling coefficient (k_p_) were 152 pC/N and 0.37, respectively, and showed the highest values at 0.12 BST. Curie temperature (T_m_) was analyzed 242 °C at 0.12 BST, the optimal composition. It was confirmed that the characteristics of 0.12 BST were excellent in all conditions. Therefore, it was confirmed that 0.12 BST is the optimal composition for (1 − x)BNT–xBST piezoelectric ceramics.

## 1. Introduction

PZT-based piezoelectric ceramics have been widely used as electro-devices due to their excellent piezoelectric properties and high stability [1,2]. However, the use of lead has begun to be regulated because the high content of lead not only hurts the environment but also harms the human body. Accordingly, research on lead-free ceramics has been actively conducted [3]. Various types of ceramics are being studied due to their excellent piezoelectric properties, including (K, Na)NbO_3_, BaTiO_3_, Bi_0.5_Na_0.5_TiO_3_, and BLSF-based ceramics, which are classified as lead-free piezoelectric ceramics [4,5,6]. Among them, Bi_0.5_Na_0.5_TiO_3_ (BNT) has a high Curie temperature T_c_ (320 °C), a remnant polarization P_r_ (38 μC/cm^2^), and a high coercive field E_c_ (7.3 kV/mm) [7]. However, due to the depolarization temperature (T_d_) that appears around 185 °C, there is a limit to its use as an actuator. In addition, leakage current is generated due to the volatilization of Bi and Na, which makes the polarization process difficult. In general, BNT materials are not easily polarized at low electric fields, so they have a higher coercive electric field than other piezoelectric ceramics. BNT piezoelectric ceramics exhibit weak thermal values at low electric fields. These large strain values are based on field-induced phase transitions. In particular, Jeong et al. demonstrated that electric field-induced phase transitions can enhance large strains for polycrystalline materials composed of large grains and diastolic matrices. Without coupling any other components, the ternary BNT system exhibits moderate piezoelectric properties. BNT has a rhombohedral perovskite structure with a piezoelectric charge coefficient of 66 pC/N and an electromechanical coupling coefficient of 21.9%. This material exhibits a high reverse piezoelectric charge factor of 174 pm/V at an electric field of 8 kV/m. However, introducing other components such as Li, Ba, Zr, and K can enhance the piezoelectric properties [8]. Therefore, it is necessary to improve the piezoelectric properties by adding an appropriate dopant [9]. To improve piezoelectric properties, many studies on BNT-based solid solutions have been conducted: such as BNT–BT, BNT–BKT, and BNT–ST [10,11,12,13,14]. BNT–BST materials have recently been studied in various works [15,16,17]. Ba_0.5_Sr_0.5_TiO_3_ (BST) is proposed as another alternative dopant. Since the unit cell volume of BST is more significant than that of BNT, it is advantageous to improve piezoelectric properties. Further, since BST (1750) has a higher permittivity than BNT (500) and has the same perovskite structure, it affects the improvement of BNT permittivity. This experiment was conducted to analyze the existing composition of BNT–BST. The advantages of the BNT–BST configuration are as follows: First, the relative permittivity of BNT–BST ceramics is slightly higher than that of BNT systems. Additionally, the piezoelectric charge coefficient of the BNT–BST system is also higher than that of the BNT systems. Finally, BNT–BST is a lead-free piezoelectric material.

In this study, BNT–BST piezoelectric ceramics were fabricated in several compositions. Previously, the piezoelectric properties of BNT–BST materials had been analyzed. However, tetragonality was analyzed through 002/200 peak XRD analysis, and the properties of piezoelectric ceramics were studied through microstructure analysis through SEM analysis. In addition, the composition was analyzed and studied in more detail compared to the existing research on BNT–BST ceramics. Therefore, this study analyzed the optimal composition of the BNT-BST ceramic through electrical properties.

## 2. Materials and Methods

(1 − x)BNT–xBST (x = 0.08, 0.10, 0.12, 0.14, 0.16) was fabricated through the conventional sintering method: solid-state reaction. Bi_2_O_3_ (99.9% purity, Sigma-Aldrich Co., Ltd., Darmstadt, Germany), Na_2_CO_3_ (99.0% purity, Sigma-Aldrich Co., Ltd., Germany), BaCO_3_ (99.0% purity, Sigma-Aldrich Co., Ltd., Milan, Italy), SrCO_3_ (99.9% purity, Sigma-Aldrich Co., Ltd., Italy), and TiO_2_ (99.9% purity, High Purity Chemicals, Tokyo, Japan) were used as raw materials. BNT was stoichiometrically weighed out of Bi_2_O_3_, Na_2_CO_3_, and TiO_2_ and ball-milled with an yttria-stabilized zirconia ball and anhydrous ethyl alcohol for 24 h. Thereafter, it was calcined at 900 °C for 2 h. BST was calcined at 800 °C for 2 h using BaCO_3_, SrCO_3_, and TiO_2_ in the same way. The calcined BNT and BST powders were weighed by 8, 10, 12, 14, and 16 mol %, mixed, and then ball-milled for 24 h. Thereafter, ethanol was dried at 120 °C, and a PVA binder was added to the sieve to a size of 100 μm to prepare a uniform powder. It was made into discs with a radius of 10 mm through single-axis press molding and sintered at 1200 °C for 3 h. The fabricated specimens were polarized for 30 min in a silicone oil bath under a DC electric field of 4 kV/mm.

The crystal structure of the fabricated BNT–BST ceramics was analyzed by X-ray diffraction (XRD; Rigaku, MiniFlex 600, Tokyo, Japan). The structure of the surface was observed through field-emission scanning electron microscopy (FE-SEM; Carl Zeiss, SIGMA 300, Oberkochen, Germany). The density of manufactured ceramics was measured by the Archimedes method. BNT–BST ceramics were polished and pasted with silver electrodes on both sides to measure the electric properties. The piezoelectric coefficient d_33_ was measured using a d_33_ meter (YE2730A, Manchester, UK). The effective permittivity coefficient (ε_r_) according to frequency was measured through an impedance analyzer (Agilent 4294A, Santa Clara, CA, USA). The electromechanical coupling coefficient was calculated through the measured resonant and antiresonant frequencies. Remnant polarization was measured under an alternating electric field of 4 kV/mm at 60 Hz—the Sawyer–Tower method [18]. To obtain the maximum dielectric permittivity temperature (T_m_), an impedance analyzer (FLUKE PM6304) was used to obtain the value when the temperature dropped.

## 3. Results and Discussion

**Figure 1:**
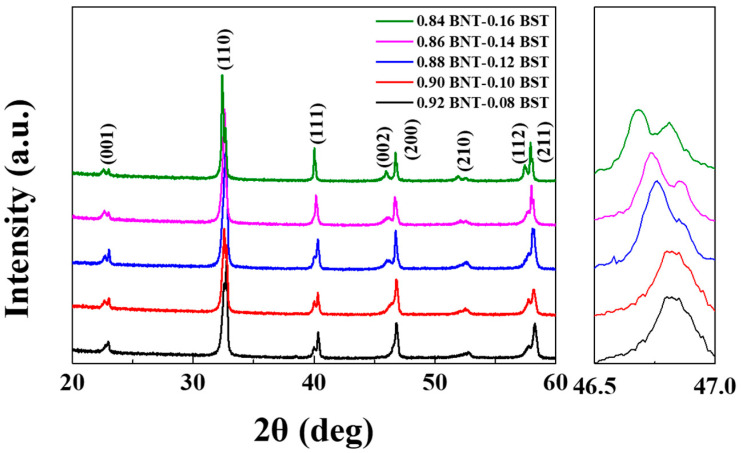
X-ray diffraction of (1 − x)BNT–xBST ceramics according to BST content.

Figure 1 shows the XRD patterns of sintered (1 − x)BNT–xBST ceramics. All compositions had a perovskite structure; a secondary phase was not present, such as a pyrochlore phase. As the BST composition increased, it was observed that the peak shifted to a lower angle. This phenomenon is because the lattice parameter increases as the ratio of Ba^2+^ (1.61 Å) and Sr^2+^ (1.44 Å) ions with relatively significant ionic radii increases. (The ionic radii of Bi^3+^ and Na^+^ are 1.40 Å and 1.39 Å, respectively) [19]. It can be inferred from the tolerance factor that small ions occupy the B-site (r < 0.87 Å) and large ions occupy the A-site (r > 0.94 Å) [20]. Considering the cation radii of Ba^2+^ (r = 1.61 Å) and Sr^2+^ (r = 1.44 Å), they tend to enter the A-site of the perovskite structure, which has some effect on the crystal lattice. At the 111 peak, around 40°, it can be seen that the split peaks merge as the composition of BST increases. In addition, it can be seen that the 002 peak around 46° is split when the BST composition is 12 mol % or more. Around x = 0.12, the phase changes from a rhombohedral structure to a tetragonal structure. The lattice constant of ceramics according to the BST composition is shown in Table 1 and was calculated by the following Equations (1) and (2) [21,22]:

(1) $$\frac{1}{d_{Rhombohedral}^{2}}=\frac{1}{a^{2}}\frac{\left(h^{2}+k^{2}+l^{2}\right)\sin^{2}a+2\left(hk+kl+lh\right)\left(\cos^{2}a+\cos a\right)}{1-3\cos^{2}a+2\cos^{3}a\;}$$

(2) $$\frac{1}{d_{Tetragonal}^{2}}=\frac{h^{2}+k^{2}}{a^{2}}+\frac{l^{2}}{c^{2}}$$

**Figure 2:**
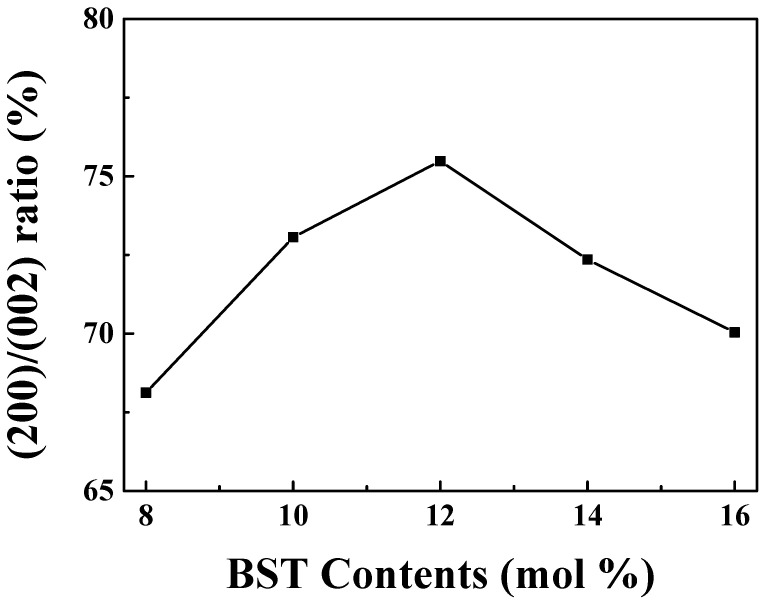
Peak ratio of (200)/(002) in the (1 − x)BNT–xBST according to BST content.

Figure 2 shows the ratio of (200)/(002) for various BST compositions. It was confirmed that the (200)/(002) ratio increased as the BST composition increased to 0.12 BST. In 0.12 BST, the ratio of (200)/(002) was confirmed to be 75.5%, and it was confirmed that the most c-axis growth was observed. In BST-based piezoelectric ceramics, as the BST content increased, the increase in the (200)/(002) ratio affected the increase in tetragonality. After that, as the content of BST increased, the (200)/(002) ratio seemed to decrease due to two split peaks [23,24]. Therefore, 0.12 BST is expected to have excellent electrical and piezoelectric properties in.

**Figure 3:**
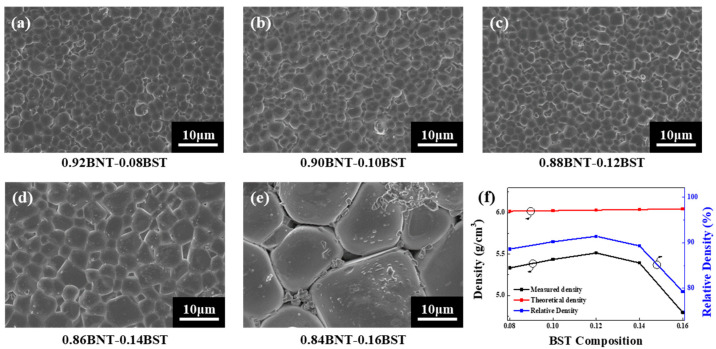
SEM images of (1 − x)BNT–xBST ceramics according to BST composition with x = (**a**) 0.08, (**b**) 0.10, (**c**) 0.12, (**d**) 0.14, and (**e**) 0.16. (**f**) Density according to composition of fabricated ceramics.

Figure 3 shows the FE-SEM images of the (1 − x)BNT–xBST ceramics. A dense crystal structure was confirmed in the range of BST composition of 0.08 ≤ x ≤ 0.12, as shown in Figure 3a–c. In general, it has been reported that ceramics having a high-density microstructure have improved electrical properties [25]. Generally, ceramics with a dense microstructure are reported to have improved electrical properties [26]. It is known that the average grain size for the composition x = 0.08–0.12 is about 2 μm [27]. The average grain size was 1.82, 1.94, and 1.99 μm in 0.08 BST, 0.10 BST, and 0.12 BST, respectively. In the composition of 0.14 BST, as shown in Figure 3d, the grains were relatively non-uniform, and the electrical properties were expected to be poor. In particular, numbers of pores were found between the grains in 0.16 BST as shown in Figure 3e. Figure 3f shows the density of the fabricated (1 − x)BNT–xBST ceramics. The theoretical density increases with the increase in BST composition because the theoretical density of BST (6.34 g/cm^3^) is relatively larger than that of BNT (5.99 g/cm^3^). As the composition of BST increased up to 0.12 BST, the density of the fabricated ceramics increased [28]. However, the density decreased at 0.14 BST, and the poorest density appeared at 0.16 BST. This represents the cracks and pores inside the ceramic identified in Figure 3e.

**Figure 4:**
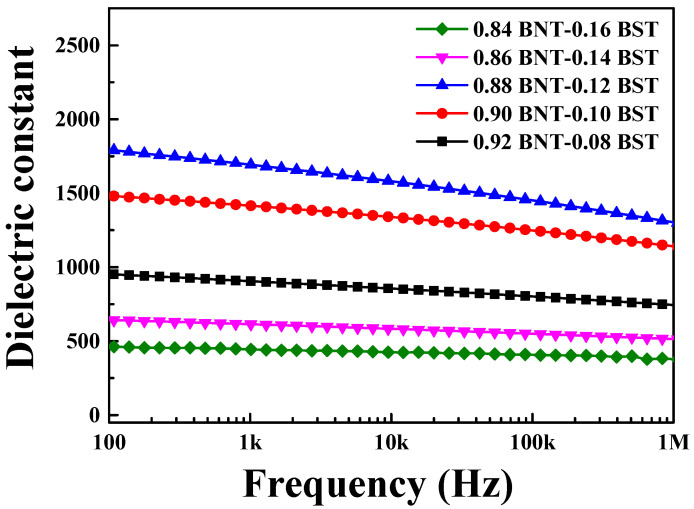
Variation in the frequency-dependent dielectric permittivity of (1 − x)BNT–xBST ceramics according to the BST content in the frequency of 100 Hz to 1 MHz.

Figure 4 shows the frequency-dependent permittivity of (1 − x)BNT–xBST ceramics at various compositions. At 1 kHz, 0.08 BST rose to 0.12 BST, and the permittivity increased from 905 to 1692, and as the BST composition increased to 0.16 BST, it was confirmed that the dielectric constant decreased to 444. Since the sintering temperature of BNT ceramics is lower than that of BST, it is not completely sintered as the composition of BST increases. It can be seen that the permittivity decreases again above 0.14 BST. Significantly, the decrease in permittivity in 0.16 BST seems to be due to the irregular grain size. This result shows that it can be expected that 0.12 BST is the best piezoelectric properties.

**Figure 5:**
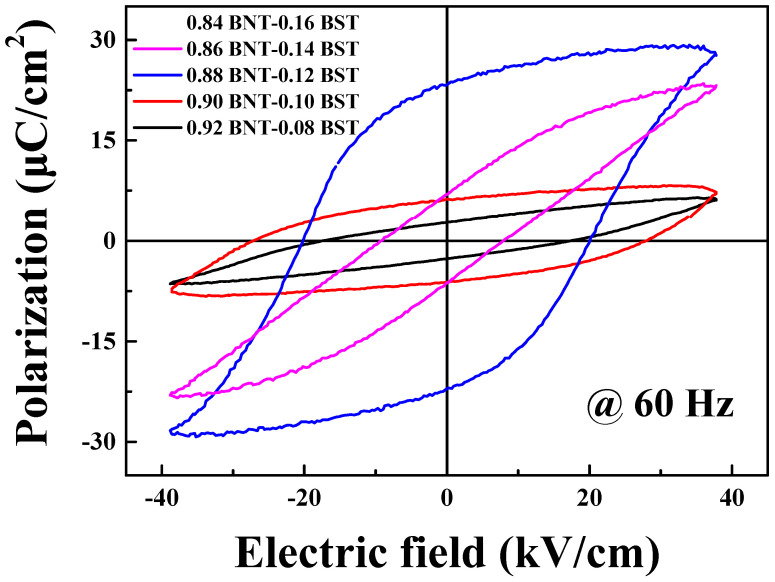
P-E hysteresis loop of (1 − x)BNT–xBST ceramics according to the BST composition.

Figure 5 shows the polarization and field hysteresis curves of (1 − x)BNT–xBST ceramics measured at a frequency of 60 Hz using the Sawyer–Tower method. It was confirmed that the remnant polarization increased as the BST composition increased to 0.12 BST [29,30]. At 0.12 BST, the saturation polarization (P_s_), remanent polarization (P_r_), and coercive electric field (E_c_) were measured as 29.2 μC/cm^2^, 23.3 μC/cm^2^, and 19.9 kV/cm, respectively, and the hysteresis loop of typical piezoelectric ceramics was confirmed. A sharp decrease in P_r_ was confirmed at 0.14 BST. In 0.16 BST, the dielectric breakdown occurred due to leakage current, which was confirmed to be caused by a number of pores between grains, as shown in Figure 3e. As can be seen in the hysteresis loop of 0.14 BST, it changes to paraelectric [31].

**Figure 6:**
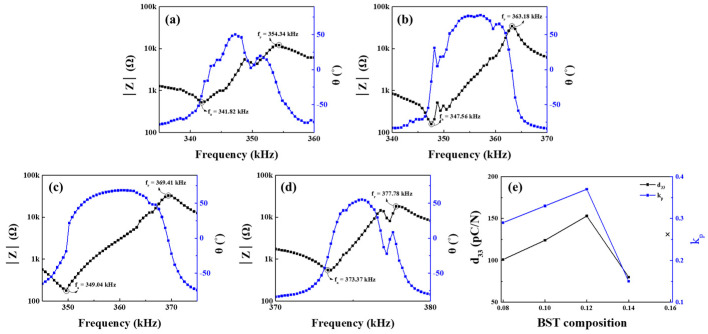
Electromechanical coupling coefficient of (1 − x)BNT–xBST ceramics according to the BST composition (x = (**a**) 0.08, (**b**) 0.10, (**c**) 0.12, and (**d**) 0.14). (**e**) Variation in piezoelectric charge constant.

Figure 6a–d shows the resonance–antiresonance points measured for each composition. It can be seen that the resonance–antiresonance frequency increases as the BST composition increases. Figure 6e shows the measured piezoelectric charge coefficient and electromechanical coupling coefficient. Since the fabricated ceramic is a disk type, the electromechanical coupling coefficient (*k_p_*) can be calculated and estimated using the following equation [32,33,34]:

(3) $$k_{p}=\sqrt{2.51\frac{f_{a}-f_{r}}{f_{a}}-{\left(\frac{f_{a}-f_{r}}{f_{a}}\right)}^{2}},$$

where *f_a_* is the anti-resonant frequency and *f_r_* is the resonant frequency. In 0.16 BST, the polarization process could not be performed due to leakage current. Figure 6e shows that the piezoelectric constant was confirmed to be excellent at 147 pC/N in 0.12 BST, and the electromechanical coupling coefficient was also confirmed with a maximum value of 0.375 for 0.12 BST. However, in 0.14 BST, d_33_ decreased to 80, while *k_p_* decreased to 0.15. As shown in Figure 3c, the composition of (1 − x)BNT–xBST ceramics was the most uniform and dense in 0.12 BST, and the highest piezoelectric properties were obtained.

**Figure 7:**
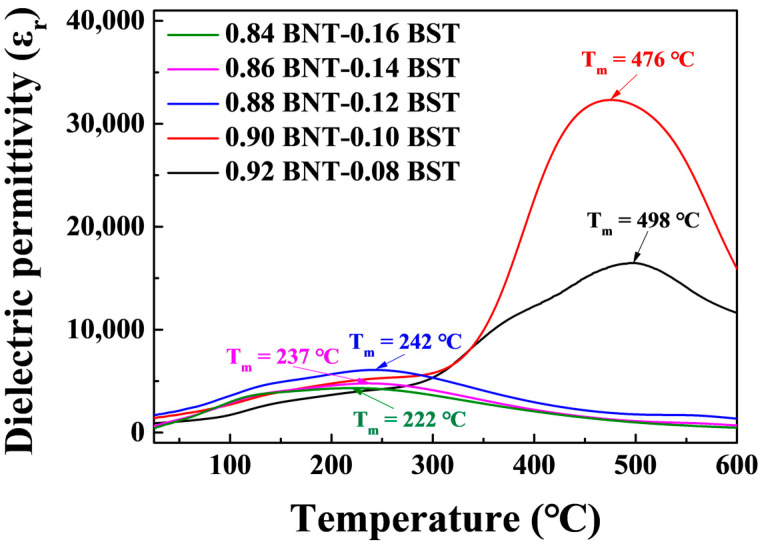
Temperature-dependent relative dielectric permittivity (ε_r_) of (1 − x)BNT–xBST ceramics sintered at 1200 °C for 3 h.

Figure 7 shows the temperature-dependent relative permittivity (ε_r_) of (1 − x)BNT–xBST (x = 0.08, 0.10, 0.12, 0.14, and 0.16) ceramics sintered at 1200 °C for 3 h. The (1 − x)BNT–xBST ceramic samples showed Curie temperatures of 498, 476, 242, 237, and 222 °C for BST contents of 0.08, 0.10, 0.12, 0.14, and 0.16%, respectively. Table 1 shows the phase change and lattice parameter change according to the BST composition. According to X. Meng et al., it is known that the Curie temperature decreases when the tolerance factor increases. The formula to calculate this factor is as follows [35]:

(4) $$\mathrm{Tolerance}\;\mathrm{factor},\;t=\frac{r_{A}+r_{O}}{\sqrt{2}\left(r_{B}+r_{O}\right)}$$

where *r_A_* is the radius of the A-site ion, *r_B_* is the radius of the B-site ion, and *r_O_* is the radius of the oxide ion at various BST compositions of (1 − x)BNT–xBST ceramics. When the content of BST is increased, the content of Ba and Sr increases, and the tolerance factor increases [36,37]. This increased tolerance factor reduced the Curie temperature. T_m_ decreased compared to low-BST compositions but remained within 200 °C for all compositions.

## 4. Conclusions

In this study, changes in properties were observed by adding 8 to 16 mol % of (Ba_0.5_Sr_0.5_)TiO_3_ to (Bi_0.5_Na_0.5_)TiO_3_-based piezoelectric ceramics. In 0.12 BST, the ratio of (200)/(002) was the highest. From there, it was confirmed that the grain size was also fine and that the crystal structure was dense. As the proportion of the BST composition increases to 12 mol %, due to the high-density microstructure, the dielectric constant was 1692, and the saturation polarization and remanent polarization were 29.2 µC/cm^2^ and 23.3 µC/cm^2^, respectively, indicating superior piezoelectric and electrical properties compared to other compositions. In addition, d_33_ was improved to 147 pC/N and k_p_ to 0.375. When the BST contents were increased, T_m_ was decreased in (1 − x)BST–xBCT ceramics. However, T_m_ was observed above 200 °C for all compositions. Therefore, lead-based piezoelectric materials are expected to be replaced by lead-free (1 − x)(Bi_0.5_Na_0.5_)TiO_3_–x(Ba_0.5_Sr_0.5_)TiO_3_ piezoelectric materials.

## Figures and Tables

**Table 1:** Lattice constant of (1 − x)BNT–xBST ceramics according to BST content.

		0.08 BST	0.10 BST	0.12 BST	0.14 BST	0.16 BST
Crystal Structure		Rhombohedral	Rhombohedral	Tetragonal	Tetragonal	Tetragonal
length	a	3.879 Å	3.879 Å	3.875 Å	3.880 Å	3.886 Å
	c			3.882 Å	3.8789 Å	3.8758 Å
Angle	α	90.2543°	90.067°	90°		

## Data Availability

The data presented in this study are available on request from the corresponding author.
